# Simultaneous determination of phenolic metabolites in Chinese citrus and grape cultivars

**DOI:** 10.7717/peerj.9083

**Published:** 2020-06-03

**Authors:** Yuan Chen, Yanyun Hong, Daofu Yang, Zhigang He, Xiaozi Lin, Guojun Wang, Wenquan Yu

**Affiliations:** 1 Institute of Agricultural Engineering and Technology, Fujian Academy of Agricultural Sciences, Fuzhou, Fujian, China; 2 Harbor Branch Oceanographic Institute, Florida Atlantic University, Fort Pierce, FL, USA; 3 Fujian Key Laboratory of Agricultural Product Food Processing (FAAS), Fuzhou, Fujian, China; 4 Hunan Provincial Key Laboratory for Biology and Control of Plant Pests, College of Plant Protection, Hunan Agricultural University, Changsha, Hunan, China; 5 Fujian Academy of Agricultural Sciences, Fuzhou, China

**Keywords:** Phenolic metabolites, Principal component analysis, Hierarchical cluster analysis, Citrus, Grape

## Abstract

**Background:**

As the major bioactive compounds in citrus and grape, it is significant to use the contents of flavonoids and phenolic acids as quality evaluation criteria to provide a better view of classifying the quality and understanding the potential health benefits of each fruit variety.

**Methods:**

A total of 15 varieties of citrus and 12 varieties of grapes were collected from Fujian, China. High-performance liquid chromatography method was used for the simultaneous determination of 17 phenolic compounds, including gallic acid, chlorogenic acid, caffeic acid, syringic acid, ρ-coumaric acid, ferulic acid, benzoic acid, salicylic acid, catechin, epicatechin, resveratrol, rutin, naringin, hesperidin, quercetin, nobiletin and tangeritin in the peels of citrus and grape cultivars. Further, the cultivars of citrus and grape were classified using principal component analysis (PCA) and hierarchical cluster analysis (HCA).

**Results:**

A thorough separation of the 17 compounds was achieved within 100 min. The tested method exhibited good linearity (the limits of detection and limits of quantification were in the range of 0.03–1.83 µg/mL and 0.09–5.55 µg/mL, respectively), precision (the relative standard deviations of repeatability were 1.02–1.97%), and recovery (92.2–102.82%) for all the compounds, which could be used for the simultaneous determination of phenolic compounds in citrus and grape. Hesperidin (12.93–26,160.98 µg/g DW) and salicylic acid (5.35–751.02 µg/g DW) were the main flavonoids and phenolic acids in 15 citrus varieties, respectively. Besides, the hesperidin (ND to 605.48 µg/g DW) and salicylic acid (ND to 1,461.79 µg/g DW) were found as the highest flavonoid and the most abundant phenolic acid in grapes, respectively. A total of 15 citrus and 12 grape samples were classified into two main groups by PCA and HCA with strong consistency.

## Introduction

Phenolic compounds are secondary metabolites widely distributed in fruits and vegetables, which are essential for the nutritional, commercial, and organoleptic qualities of the fruit. The growing interest in phenolic metabolites is mainly due to their biological activities, such as anti-inflammation, antioxidant, antimicrobial and anticancer (Gu et al., 2019; Lou & Ho, 2017; Rasines-Perea & Teissedre, 2017). Observational studies have shown that polyphenols significantly reduced the risk of hypertension and cardiovascular disease (CVD) (Rasines-Perea & Teissedre, 2017).

Grapes and citrus contain a considerable amount of different phenolic compounds in peels, leaves, pulp and seeds, contributing to high nutritional value (De Paula Menezes Barbosa, Ruviaro & Macedo, 2018; Ernawita et al., 2017; Liew et al., 2018). As a major bioactive compound in citrus and grape, it is significant to use phenolic compound content as a quality/nutritional evaluation criteria for different varieties of these fruits grown in different environmental conditions to provide a quantifiable classification of the quality and potential health benefits of each fruit variety. Besides, phenolic acids and flavonoids are the most abundant phenolic compounds in grapes and citrus (Da Silva Padilha et al., 2017; Wang et al., 2017).

Several methods have been reported for simultaneous determination of flavonoids and phenolic acids in citrus or grape. Wang et al. (2019) identified 254 flavonoid metabolites including eight isoflavone, 21 flavanone, 24 anthocyanins, 39 flavonol, 147 flavone and 15 polyphenol in the peels of five citrus varieties by UPLC-ESI-MS/MS method. Wang et al. (2017) investigated the flavonoids in the peels of 35 citrus varieties by li quid chromatography combined with electrospray ionization mass spectrometry (LC-ESI-MS/MS) and ultra-performance liquid chromatography combined with diode array detector (UPLC-DAD). In addition, RP-High-performance liquid chromatography (HPLC)/DAD was applied to determine the flavonoids and phenolic acids in grapes (Da Silva Padilha et al., 2017).

Many studies have investigated the analysis and detection of phenolic compounds in citrus and grape varieties, but little is known to the simultaneous determination of flavonoids and phenolic acids in the peel extracts of citrus and grape. Considering the similar phenolic groups in citrus and grapes, we hypothesized that a quantification and identification method could be established to simultaneously determine several phenolic compounds for citrus and grape. Therefore, this study aimed to develop a novel, direct, optimized and validated method for the simultaneous identification and quantification of phenolic compound in citrus and grape peel extracts using HPLC-DAD with a fast sample preparation. The study was also designed to apply principal component analysis (PCA) and hierarchical cluster analysis (HCA) as exploratory methods to analyze the phenolic compound profile, and to understand the differences between varieties of citrus and grape.

## Materials and Methods

### Chemicals

All 17 standard compounds of gallic acid, chlorogenic acid, caffeic acid, syringic acid, ρ-coumaric acid, ferulic acid, benzoic acid, salicylic acid, catechin, epicatechin, rutin, naringin, hesperidin, quercetin, resveratrol, nobiletin and tangeritin were purchased from Sigma (St. Louis, MO, USA), and were certified as >98% purity. The compounds were prepared in methanol-dimethyl sulfoxide (DMSO) (v/v, 50:50), and stored at −18 °C for 2 weeks. During development of the HPLC-DAD method, working standard solutions were obtained by dilution of individual phenolic stock solutions with DMSO (v/v, 50:50) solution to obtain five different concentrations for calibration curves. All the other reagents of analytical grade were bought from Sinopharm Chemical Reagent Co., Ltd. (Shanghai, China).

### Fruit materials

Fifteen varieties of citrus listed in Table 1 were harvested from trees in local farms at Pudang, Fujian, China, between October 2012 and January 2013. Twelve varieties of grapes were collected from Jianou, Fujian, China (Table 1). Following this, the peel of each fruit was separated from the edible portion, and then freeze-dried, ground and finally stored at −20 °C prior to use.

**Table 1:** Citrus and grape cultivars used in this study.

Number	Scientific name (Latin name)	Chinese name	Abbreviation
Citrus			
1	*Citrus grandis*(L.) *Osbeck “Duweiwendan”*	Duweiwendan	DWWD
2	*Citrus grandis*(L.) *Osbeck “Hongmianmiyou”*	Hongmianmiyou	HMMY
3	*Citrus grandis*(L.) *Osbeck “Hongroumiyou”*	huangjinyou	HRMY
4	*Citrus grandis*(L.) *Osbeck “Guanximiyou”*	Guanximiyou	GXMY
5	*Citrus grandis*(L.) *Osbeck “Huangroumiyou”*	Huangjinyou	HJY
6	*Citrus paradisi × Citrus* sp. *“Huyou”*	Huyou	HY
7	*Citrus paradisii* Macf.	Putaoyou	PTY
8	*Citrus grandis* (L.) *Osbeck “Pingshanyou”*	Pingshanyou	PSY
9	*Citrus nobilis* Lour. *“Gonggan”*	Gonggan	GG
10	*Citrus chuana* Hort. *ex Tseng’*	Wenzhoumigan	WZMY
11	*Citrus nobilis* Lour.	Nanfengmiju	NFMJ
12	*Citrus. reticulata × C. sinensis*	Maogujucheng	MGJC
13	*Citrus sinensis Osbeck*	Niuheerqicheng	NHEQC
14	*Citrus limon* (L.) *Bur*	Youlike	YLK
15	*Citrus reticulata* Blanco	Fuju	FJ
Grape			
1	*Vitis vinifera* L. *× V. labrusca*L.	Xiahei	XH1
2	*Vitis vinifera* L. *× V. labrusca*L.	Xiahei	XH2
3	*Vitis vinifera* L. *× V. labrusca*L.	Xiahei	XH3
4	*Vitis vinifera* L.	Biankou	BAK
5	*Vitis vinifera* L.	Dongxian	DX
6	*Vitis vinifera* L. *× V. labrusca*L.	Meiman	MM
7	*Vitis vinifera* L.	Hongru	HR
8	*Vitis vinifera* L. *× V. labrusca*L.	Huangmi	HM
9	*Vitis vinifera* L.	Baijixin	BJX
10	*Vitis vinifera* L. *× V. labrusca*L.	Yesheng	YS
11	*Vitis vinifera* L. *× V. labrusca*L.	Jufeng	JF
12	*Vitis vinifera* L. *× V. labrusca*L.	Jumeigui	JMG

### Sample preparation

A mixture of 0.1 g peel sample and 1.0 mL methanol-DMSO (50:50 v/v) was placed in a centrifuge tube, stirred for 10 min at room temperature, and centrifuged at 9,000×*g* rpm for 15 min at 4 °C. The resulting residues were extracted twice with the same extraction solvent (one mL). All the supernatants were combined and made up to 5.0 mL with methanol. All the sample solutions were filtered through a 0.45 μm membrane filter before use.

### Simultaneous separation and quantitative analysis of phenolic metabolites

Phenolic metabolites were separated by an Agilent 1260/6410 HPLC system (Agilent, Santa Clara, CA, USA) consisting of an L-2455 diode array detector (DAD) and LiChrospher^®^100RP-18e column (4.0 × 250 mm, 5 μm particle size, Merck, Darmstadt, Germany). The mobile phases consisted of solution A (water with 2% (v/v) acetic acid) and solution B (100% acetonitrile); gradient elution was performed as follows: 4% B in the first 5 min, increased to 8% at 18 min; kept isocratically with 8% B for the following 22 min, then increased to 30% B at 75 min, 50% B within next 15 min followed by another isocratic step with 50% B for 5 min, and linear gradient back to 4% B in 5 min. The flow rate was 0.8 mL/min and the total run time was 100 min. 10 µL of each solution was injected for analysis. The detector was monitored at 280 nm and 330 nm. Electrospray mass spectrometry was performed in the positive ion mode; dry gas, N2, flow rate, 8 L/min; pressure, 40 psi; temperature. 250 °C; capillary voltage. 4,500 v; and fragmentor voltage, 135 v. MS2 scans ranged from 50 m/z to 2,000 m/z. Quantification of each compound was carried out by an external standard method using calibration curves. Calibration linear curve for each compound was constructed by regression peak area (*y*) and concentration (*C*). At least five spots within the linear range were used to obtain the regression curve.

### Limit of detection and limit of quantification

The Limit of detection (LOD) and limit of quantification (LOQ) for each compound were calculated by repeat injections (*n* = 10) of a mixture of standard solution at a known concentration. The LOD and LOQ values were then calculated based on the standard deviation (SD) of the response and the slope (Bressolle, Bromet-Petit & Audran, 1996), as shown in Eqs. (1) and (2),

(1) $${\rm LOD} = \displaystyle{{3.3\sigma } \over S}$$

(2) $${\rm LOQ} = \displaystyle{{10\sigma } \over S}$$

where σ is the SD of the response, *S* is the slope of the calibration curve.

### Repeatability

The repeatability was evaluated by five consecutive injections of a mixture of the standard solution in 1 day. The relative standard deviations (RSDs) of peak area for each compound were used to evaluate the repeatability.

### Recovery rate

The recovery was studied by calculating the mean recoveries of the analysts using the standard addition method (Francisco & Resurreccion, 2009). The reference standards were added at three different concentration levels (approximately equivalent to 0.8, 1.0 and 1.2 times of the known concentration of the matrix) with three parallels at each level. Then, the solutions were extracted and analyzed. The recovery was calculated using the Eq. (3):

(3) $${\rm Recovery\; }\left( {\rm \% } \right){\rm \; } = {\rm \; }\displaystyle{{{\rm Amount\; determined\; } - {\rm \; Original\; amount}} \over {{\rm Amount\; spiked}}}{\rm \; } \times {\rm \; }100{\rm \% }$$

### Statistical analysis

All the samples were prepared and analyzed in triplicate. All data were expressed as the mean ± SD. Multiple group comparison was done by ANOVA and Tukey’s test (*P* < 0.05) using SAS 9.4 (NC, USA). PCA and HCA were performed with IBM SPSS version 22 (NY, USA) to classify and discriminate among the citrus and grape cultivars.

## Results

### HPLC method validation

#### HPLC chromatographic profiles

**Figure 1:**
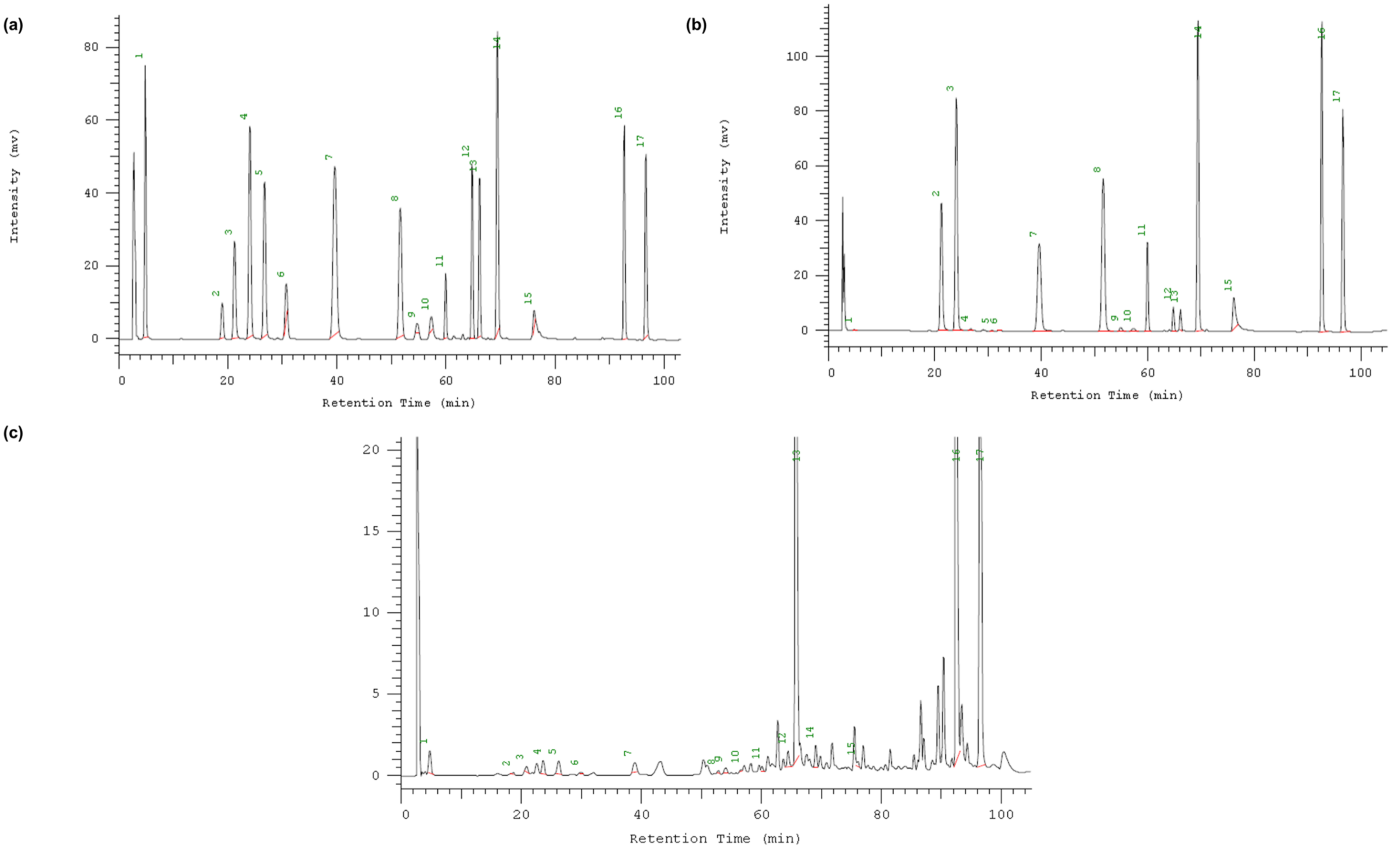
HPLC chromatograms with the compounds. (A) standard solutions; (B) citrus representative sample-GG sample in Table 1; (C) grape representative sample—JF sample in Table 1. Peak numbers: (1) Gallic acid; (2) Catechin; (3) chlorogenic acid; (4) caffeic acid; (5) syringic acid; (6) epicatechin; (7) ρ-Coumaric acid; (8) ferulic acid; (9) benzoic Acid; (10) salicylic acid; (11) rutin; (12) naringin; (13) hesperidin; (14) resveratrol; (15) quercetin; (16) nobiletin; (17) Tangeretin.

The chromatographic profiles of the standard solutions and the sample representatives monitored at 280 nm/330 nm were shown in Fig. 1. The HPLC condition obviously separated these compounds in the standard solutions and the sample representatives within the running time. Then the method was validated.

#### Linearity, LOD and LOQ

All results of method validation are summarized in Table 2. The linearity of each compound was created in a wide concentration ranging from 0.09 to 100 mg/mL for different compounds. All the correlation coefficients (*r*^2^) of the calibration curves were above 0.9976, indicating good linearity of the calibration curves. The LODs and LOQs of the 17 compounds were in the range of 0.03–1.83 µg/mL and 0.09–5.55 µg/mL, respectively, suggesting good sensitivity of the developed method.

**Table 2:** Regression equation, correlation coefficient, linear range, limit of detection (LOD) and limit of quantification (LOQ) of the phenolic compositions.

Compound	Wavelength (nm)	m/z	Retention time (min)	Linearity		Linear range (μg/ml)	LOD (μg/ml)	LOQ (μg/ml)	Repeatability (*n* = 5)	Recovery (%)
				Calibration equations^a^	Correlation coefficient					
Gallic acid	280	170	4.93	*y* = 16486.96 *x* − 1935.75	0.9999	0.11–100	0.03	0.09	1.11	101.58
Catechin	280	290	19.2	*y* = 4362.63 *x* − 1075.23	0.9998	0.43–100	0.13	0.39	1.18	92.20
Chlorogenic acid	280	354	21.61	*y* = 11217.41 *x* − 3852.84	0.9997	0.18–100	0.05	0.15	1.66	95.56
Caffeic acid	330	180	24.29	*y* = 23730.91 *x* − 4696.96	0.9999	0.09–100	0.03	0.09	1.28	98.48
Syringic acid	280	198	27.12	*y* = 20842.32 *x* − 3904.67	0.9999	0.40–100	0.12	0.36	1.02	98.89
Epicatechin	280	290	30.99	*y* = 8484.05 *x* − 2672.05	0.9998	2.5–100	0.72	2.18	1.41	99.58
ρ-Coumaric acid	330	164	40.23	*y* = 36409.07 *x* − 5490.19	0.9999	0.86–100	0.26	0.79	1.24	99.22
Ferulic acid	330	194	52.16	*y* = 21047.21 *x* − 3361.75	0.9999	0.27–100	0.08	0.24	1.53	102.82
Benzoic acid	280	123	54.95	*y* = 3309.42 *x* − 99.98	0.9999	0.53–100	0.16	0.48	1.49	93.60
Salicylic acid	330	138	57.21	*y* = 4286.10 *x* − 2290.45	0.9991	2.5–100	1.19	3.61	1.90	94.30
Rutin	280	611	60.16	*y* = 4643.32 *x* + 1353.07	0.9976	0.52–100	0.15	0.45	1.44	92.20
Naringin	280	581	64.97	*y* = 12259.00 *x* − 1724.35	0.9999	0.38–100	0.11	0.33	1.22	96.34
Hesperidin	280	611	66.34	*y* = 11424.82 *x* − 2542.83	0.9998	0.50–100	0.15	0.45	1.20	98.54
Resveratrol	330	228	69.80	*y* = 26014.88 *x* − 4569.33	0.9999	0.22–100	0.07	0.21	1.37	96.09
Quercetin	330	302	76.52	*y* = 4515.99 *x* − 16433.83	0.9991	2.5–100	0.13	0.39	1.97	94.74
Nobiletin	330	403	93.08	*y* = 15335.05 *x* − 2413.05	0.9999	0.80–200	0.24	0.73	1.20	100.39
Tangeritin	330	373	97.09	*y* = 15914.66 *x* + 12562.01	0.9994	2.5–200	1.83	5.55	1.55	96.27

**Note:**

a *y* is peak area and *x* is concentration of each standard (μg/mL).

#### Precision

The precision of the method was assessed by repeatability, which was evaluated by five consecutive injections of a sample representative (GG as citrus representative and JF as grape representative) in one day. RSDs of peak area for each compound were used to evaluate repeatability.

As indicated in Table 2, the RSDs of repeatability were 1.02–1.97%, which were less than 3.5% for all the compounds, revealing good precision of this method.

#### Recovery

The recoveries of all the 17 compounds were in the range of 92.2–102.82%, with RSD values less than 5%, indicating that the established method was accurate enough for the determination of the 17 compounds in citrus and grape.

### Distribution of the 16 phenolic components in 15 citrus cultivars

A total of eight flavonoids, including rutin, naringin, hesperidin, quercetin, nobiletin, tangeritin, catechin and epicatechin (Tables 3A and 4A) were used to represent the flavonoid compositions in citrus and grape.

**Table 3:** The contents of flavonoids and phenolic acid in the peels of the 15 different citrus cultivars analyzed in this study (µg/g DW)*.

(A) The contents of flavonoids in the peels of the 15 different citrus cultivars analyzed in this study (μg/g DW)*								
	Naringin	Hesperidin	Rutin	Quercetin	Nobiletin	Tangeritin	Catechin	Epicatechin
DWWD	8061.35 ± 208.21 bc	369.68 ± 4.64 g	107.41 ± 6.33 bc	144.4 ± 11.19 d	1.14 ± 0.18 c	1.21 ± 0.09 c	ND^#^ g	ND f
HMMY	8203.71 ± 65.82b	12.93 ± 1.37 g	101.45 ± 2.92 bc	148.92 ± 4.16 d	1.75 ± 0.07 c	17.96 ± 1.35 c	22.66 ± 0.63f	9.29 ± 0.29 c
HRMY	8113.34 ± 93.92b	32.93 ± 1.45 g	13.42 ± 1.82 e	73.41 ± 2.07 d	6.76 ± 0.12 c	55.27 ± 2.75 c	16.61 ± 0.35 f	ND f
GXMY	5943.69 ± 164.05d	309.18 ± 4.84 g	79.87 ± 2.00 cd	47.28 ± 6.71 d	2.68 ± 0.04c	0.79 ± 0.07 c	54.38 ± 3.85 d	ND f
HJY	6369.13 ± 116.15 d	187.01 ± 13.51 g	79.34 ± 2.07 cd	82.9 ± 1.98 d	0.71 ± 0.07 c	1.48 ± 0.08 c	97.93 ± 0.33 b	0.36 ± 0.02 f
HY	7651.39 ± 201.4c	545.24 ± 18.8 fg	34.5 ± 2.27 de	16.51 ± 0.55 d	55.29 ± 1.94 c	42.65 ± 1.04 c	7.61 ± 1.11 g	ND f
PTY	3608.76 ± 113.99e	385.23 ± 12.37 g	1801.38 ± 57.7 a	25.66 ± 2.81 d	76.99 ± 3.02 c	74.66 ± 4.22 c	100.79 ± 5.12 b	ND f
PSY	97.87 ± 2.42f	15973.95 ± 486.26	38.36 ± 1.42 de	498.68 ± 654.92 cd	540.66 ± 739.99	513.34 ± 696.48	22.84 ± 0.77 f	ND f
GG	35.31 ± 2.29 f	9749.17 ± 225.89 d	77.27 ± 2.39 cd	736.27 ± 15.85 c	899.41 ± 21.85 b	769.76 ± 9.75 b	22.02 ± 1.67 f	21.94 ± 0.78 a
WZMY	49.33 ± 0.53 f	26160.98 ± 769.9 a	132.85 ± 2.84 b	361.73 ± 11.39 cd	2661.66 ± 62.01 a	2243.16 ± 51.71 a	ND g	20.83 ± 0.76 b
NFMJ	48.38 ± 6.77 f	20121.89 ± 506.55 b	79.11 ± 3.6 cd	1300.86 ± 22.31 b	76.64 ± 4.08 c	41.16 ± 1.09 c	69.44 ± 3.66 c	ND f
MGJC	19.71 ± 0.43 f	272.85 ± 10.87 g	17.04 ± 0.58 e	1816.04 ± 9.25 a	889.37 ± 12.53 b	309.47 ± 9.42 bc	105.02 ± 0.86 b	5.16 ± 0.44 d
NHEQC	16140.43 ± 414.24 a	1363.42 ± 87.76 f	26.32 ± 3.13 e	487.26 ± 4.58 cd	2.22 ± 0.13 c	ND c	33.21 ± 1.33 e	ND f
YLK	253.53 ± 4.41 f	7992.9 ± 67.24 e	31.07 ± 0.69 e	4.07 ± 0.26 d	ND c	35.44 ± 1.62 c	152.18 ± 5.79 a	ND f
FJ	28.15 ± 0.46 f	8282.28 ± 171.29 e	9.89 ± 0.34 e	178.12 ± 4.57 d	703.75 ± 9.81 b	504.91 ± 12.6 bc	53.11 ± 2.34 d	3.69 ± 0.17 e
Total	49847.07	106536.65	2629.28	5922.11	5919.03	4611.26	757.8	61.27

(B) The phenolic acid contents in the peels of the 15 different citrus cultivars analyzed in this study (µg/g DW)*								
	Gallic acid	Chlorogenic acid	Caffeic acid	Syringic acid	ρ-Coumaric acid	Ferulic acid	Benzoic acid	Salicylic acid
DWWD	ND d	22.83 ± 1.12 d	8.44 ± 0.96 d	0.89 ± 0.16 gh	ND^#^ f	6.21 ± 0.76 c	54.31 ± 2.33 c	6.28 ± 0.09 i
HMMY	ND d	9.29 ± 1.37 g	9.84 ± 0.25 cd	1.2 ± 0.05 g	16.67 ± 0.92 c	1.61 ± 0.06 fgh	82.36 ± 1.44 b	22.00 ± 0.74 h
HRMY	0.85 ± 0.01 cd	20.14 ± 1.01 e	11.27 ± 0.41 c	4.87 ± 0.55 e	10.25 ± 0.16 d	4.5 ± 0.12 de	16.5 ± 0.77 e	15.64 ± 1.03 hi
GXMY	1.35 ± 0.14 c	3.53 ± 0.03 h	8.17 ± 1.03 d	0.94 ± 0.10 gh	0.28 ± 0.02 f	1.14 ± 0.12 gh	5.86 ± 0.15 h	5.37 ± 0.22 i
HJY	1.48 ± 0.08 c	1.35 ± 0.21 hi	5.13 ± 0.07 e	2.89 ± 0.08 f	0.38 ± 0.02 ef	0.85 ± 0.04 h	4.15 ± 0.17 h	11.43 ± 1.11 hi
HY	ND d	19.18 ± 0.52 e	2.48 ± 0.08 f	9.83 ± 0.75 d	ND f	2.6 ± 0.44 fg	4.23 ± 0.57 h	12.21 ± 1.99 hi
PTY	ND d	42.97 ± 1.00 b	18.85 ± 1.64	4.82 ± 0.16 e	0.79 ± 0.07 ef	2.15 ± 0.18 fgh	21.54 ± 0.79 d	222.38 ± 9.08 c
PSY	ND d	25.63 ± 0.88 c	2.41 ± 0.37 f	ND h	34.8 ± 0.42 b	28.99 ± 1.21 b	3.09 ± 0.07h	751.02 ± 12.07 a
GG	8.92 ± 0.34 b	1.73 ± 0.37 hi	13.26 ± 0.41 b	16.13 ± 0.42 b	0.54 ± 0.05 ef	3.17 ± 0.40 ef	13.47 ± 0.45 ef	12.77 ± 1.82 hi
WZMY	ND d	52.23 ± 1.00 a	ND g	ND h	ND f	2.91 ± 0.22 ef	5.32 ± 0.93 h	170.17 ± 1.69 e
NFMJ	ND d	9.63 ± 0.37 g	ND g	ND h	0.76 ± 0.05 ef	5.32 ± 0.09 cd	5.22 ± 0.18 h	202.23 ± 6.24 d
MGJC	ND d	9.54 ± 0.59 g	13.96 ± 0.98 b	14.74 ± 0.5 c	1.29 ± 0.03 e	0.71 ± 0.04 h	11.66 ± 0.49 fg	360.02 ± 10.6 b
NHEQC	ND d	22.78 ± 0.25 d	8.27 ± 0.36 d	ND h	43.94 ± 0.75 a	2.18 ± 0.12fgh	163.37 ± 2.55 a	45.1 ± 1.87 g
YLK	ND d	0.42 ± 0.04 i	2.15 ± 0.2 f	31.18 ± 0.2 a	0.26 ± 0.01 f	30.57 ± 0.93 ab	12.31 ± 0.17 fg	65.56 ± 0.85 f
FJ	50.16 ± 1.53 a	14.79 ± 0.77 f	9.78 ± 0.43 cd	15.35 ± 0.87 bc	0.4 ± 0.01 ef	31.56 ± 1.05 a	9.14 ± 0.4 g	19.13 ± 0.41 hi
Total	62.76	256.04	114.01	102.84	110.36	124.47	412.53	1921.31

**Notes:**

* Values are means ± standard deviation of means from three repeats. Different letters in each column indicate significant difference between samples (*P* < 0.05).

# Not detectable.

**Table 4:** The contents of flavonoids and phenolic acid in the skin of the 12 grape cultivars analyzed in this study (µg/g DW)*.

(A) The contents of flavonoids in the skin of the 12 grape cultivars analyzed in this study (µg/g DW)*								
	Naringin	Hesperidin	Rutin	Quercetin	Nobiletin	Tangeritin	Catechin	Epicatechin
XH1	1.48 ± 0.12 g	159.6 ± 12.32 e	93.79 ± 5.08 e	270.00 ± 8.90 b	25.75 ± 2.63 d	5.25 ± 0.41 de	57.33 ± 2.69 e	52.12 ± 3.75 e
XH2	1.63 ± 0.05 g	241.02 ± 4.45 c	201.95 ± 3.72 c	339.43 ± 8.58 a	0.3 ± 0.02 g	11.15 ± 0.53 c	40.58 ± 1.22 f	98.55 ± 4.81 d
XH3	26.37 ± 0.87 c	351.92 ± 2.84 b	51.36 ± 2.15 f	320.77 ± 15.53 a	0.36 ± 0.02 g	3.41 ± 0.13 e	97.44 ± 1.31 d	102.55 ± 4.88 d
BAK	50.20 ± 2.56 b	30.38 ± 1.95 gh	910.12 ± 25.96 a	ND^#^ f	2.45 ± 0.11 fg	101.13 ± 2.86 a	243.08 ± 9.44 b	53.83 ± 2.13 e
DX	8.97 ± 0.13 de	209.94 ± 13.04 d	17.06 ± 0.46 gh	28.85 ± 2.01 e	6.91 ± 0.88 f	100.13 ± 5.35 a	31.31 ± 1.81 f	ND g
MM	12.99 ± 1.52 d	103.82 ± 5.58 f	326.73 ± 3.51 b	ND f	60.19 ± 4.33 a	25.72 ± 1.05 b	132.01 ± 1.68 c	206.2 ± 6.95 a
HR	106.43 ± 5.26 a	ND i	4.41 ± 0.39 h	74.57 ± 2.55 d	46.22 ± 3.34 b	3.08 ± 0.2	2.15 ± 0.21 g	37.00 ± 1.26 f
HM	8.36 ± 0.62 def	33.84 ± 2.88g	95.93 ± 2.01 e	6.90 ± 0.27 f	37.73 ± 1.05 c	ND e	322.89 ± 4.77 a	117.59 ± 7.51 c
QT	3.71 ± 0.53 efg	10.36 ± 1.13 i	162.47 ± 1.07 d	ND f	15.75 ± 0.47 e	26.26 ± 2.39 b	34.65 ± 1.62 f	29.81 ± 0.97 f
YS	3.57 ± 0.49 fg	13.17 ± 0.75 hi	105.11 ± 6.67 e	ND f	ND g	10.85 ± 0.85 cd	87.3 ± 5.09 d	36.26 ± 1.69 f
JF	26.37 ± 0.87 c	351.92 ± 2.84 b	51.36 ± 2.15 f	320.77 ± 15.53 a	0.36 ± 0.02 g	3.41 ± 0.13 e	97.44 ± 1.31 d	102.55 ± 4.88 d
JMG	2.53 ± 0.10 g	605.48 ± 5.53 a	33.02 ± 2.02 fg	194.80 ± 3.08 c	0.51 ± 0.01 g	3.41 ± 0.24 e	56.79 ± 2.36 e	153.12 ± 6.97 b
Total	252.62	2111.46	2053.31	1556.07	196.54	293.79	1202.97	989.57

(B) The phenolic acid contents in the skin of the 12 grape cultivars analyzed in this study (µg/g DW)*								
	Gallic acid	Chlorogenic acid	Caffeic acid	Syringic acid	ρ-Coumaric acid	Ferulic acid	Benzoic acid	Salicylic acid
XH1	2.36 ± 0.13 d	33.58 ± 0.67 c	ND c	0.69 ± 0.06 f	ND f	18.62 ± 0.77 a	20.77 ± 0.79d	142.4 ± 5.83 d
XH2	7.89 ± 0.31 a	43.77 ± 0.67 b	3.46 ± 0.09 b	1.6 ± 0.08 e	0.67 ± 0.04 e	1.79 ± 0.06 e	4.95 ± 0.56 f	16.73 ± 1.24 h
XH3	3.52 ± 0.32 b	35.74 ± 0.54 c	3.81 ± 0.09 b	2.89 ± 0.17 c	8.72 ± 0.31 a	1.58 ± 0.07 ef	22.53 ± 0.91 d	11.33 ± 0.49 hi
BAK	8.1 ± 0.2 a	41.76 ± 1.84b	6.56 ± 0.36 a	ND g	2.19 ± 0.05 d	3.71 ± 0.37 d	3.57 ± 0.30 fg	39.73 ± 0.35 g
DX	ND f	231.28 ± 3.79 a	6.68 ± 0.23 a	6.68 ± 0.23 a	ND f	3.91 ± 0.07 d	13.07 ± 0.22 e	446.34 ± 7.08 f
MM	2.98 ± 0.27 bc	25.11 ± 0.64 e	3.61 ± 1.21	ND g	5.68 ± 0.13 b	5.22 ± 0.06 c	63.75 ± 1.00 a	892.46 ± 5.58 b
HR	ND f	30.17 ± 3.32 cde	ND c	ND g	ND f	ND g	37.43 ± 1.45 c	ND
HM	2.17 ± 0.18 d	35.37 ± 1.6 c	2.92 ± 0.06 b	2.11 ± 0.21 d	ND f	1.24 ± 0.03 ef	ND g	69.66 ± 1.43 f
QT	2.5 ± 0.07 cd	26.92 ± 0.78 de	2.89 ± 0.06 b	ND g	ND f	0.85 ± 0.04 f	2.7 ± 0.34 fg	16.51 ± 1.13 h
YS	ND f	32.49 ± 3.12 cd	ND c	0.76 ± 0.06 f	3.32 ± 0.04 c	ND g	3.51 ± 0.08 fg	102.81 ± 5.32 e
JF	3.52 ± 0.32 b	35.74 ± 0.54 c	3.81 ± 0.01 b	2.89 ± 0.17 c	8.72 ± 0.31 a	1.58 ± 0.07 ef	22.53 ± 0.91 d	11.33 ± 0.49 hi
JMG	1.05 ± 0.14 e	13.46 ± 0.64 f	0.41 ± 0.01 c	6.14 ± 0.06 b	2.3 ± 0.04 d	7.43 ± 0.27 b	54.59 ± 3.57 b	1461.79 ± 12.58 a
Total	34.08	585.39	34.44	23.77	31.61	45.93	249.39	3211.08

**Notes:**

* Values are means ± standard deviations of means from three repeats. Different letters in each column indicate significant difference between samples (*P* < 0.05).

# Not detectable.

#### Flavonoid composition

Hesperidin was the primary flavonoid compound in all of the citrus samples with concentration from 12.93 to 26160.98 µg/g dry weight (DW) among the 15 citrus varieties (Table 3A). While catechin and epicatechin were not major compounds. Epicatechin was under the detection limit in nine citrus samples (Table 3A). Specifically, Wenzhoumigan had the highest content of hesperidin, followed by Nanfengmiju, both of which had far higher hesperidin than other cultivars (Table 3A). In addition, nobiletin and tangeritin were abundant in Wenzhoumigan, but were not major components in most of other citrus cultivars. Hongmianmiyou had the highest content of narigin, and Maogu jucheng had the highest content of quercetin. Rutin presented higher concentration in Putaoyou (14–134 times) than the other citrus cultivars. These results provide insight into specific citrus sources for different flavonoids.

#### Phenolic acid compounds

The concentrations of phenolic acids were lower than those of flavonoids (Tables 3B vs. 3A). As shown in Table 3B, salicylic acid was the most dominant extractable phenolic acid, with content varying from 5.35 to 751.02 µg/g DW in the samples. The content of salicylic acid was the highest in Pingshanyou, which was 2–139 times higher than that of other samples. Benzoic acid was the second highest phenolic acid of all the citrus cultivars, ranging from 3.09 to 163.37 µg/g DW, which was the most abundant phenolic acid in Niuheerqicheng. The content of other phenolic acids was below 50 µg/g DW or the detection limit. Especially the gallic acid was only distributed in HRMY, GCMY, HJY, GG and FJ cultivars.

### Distribution of the 16 components in 12 grape cultivars

#### Flavonoid composition

The content and variation of each flavonoid compound in grape cultivars is summarized in Table 4A. Overall, the contents of flavonoids in the peels of grapes were lower than those in citrus (Tables 3A vs. 4A). However, similar to the flavonoid distribution in citrus, considerable differences in content of flavonoid compounds were detected among grape varieties. One obvious example is the rutin content in the three Xiahei samples. The concentration of rutin varied from 51.36 µg/g DW in Xiahei3 to 201.95 µg/g DW in Xiahei2. Among the eight flavonoid compounds, hesperidin was the most abundant, followed by rutin, quercetin, catechin and epicatechin (Table 4A).

The variation of these flavonoids in different grape varieties is shown in Table 4A. Rutin was the most abundant compound in Biankou. Quercetin was the most abundant flavonoid in Xiahei1 and Xiahei2, whereas the concentration of hesperidin was ranking as No. 1 in Xiahei3. Hesperidin was also the most dominant compound in Biankou, Yesheng, Jufeng and Jumeigui cultivars (Table 4A).

#### Phenolic acid compounds

Similar with citrus, salicylic acid was also the most abundant phenolic acid in grape (Tables 3B vs. 4B). The content of salicylic acid in Jumeigui reached 1,461.79 µg/g DW, while it was below detection limit in Hongru (Table 4B). Chlorogenic acid was the second abundant phenolic acid (585.39 µg/g DW in total), followed by benzoic acid (249.39 µg/g DW in total). The total amount of other acids was quite similar, which was below 50 µg/g DW.

### Classification of citrus and grape cultivars

**Figure 2:**
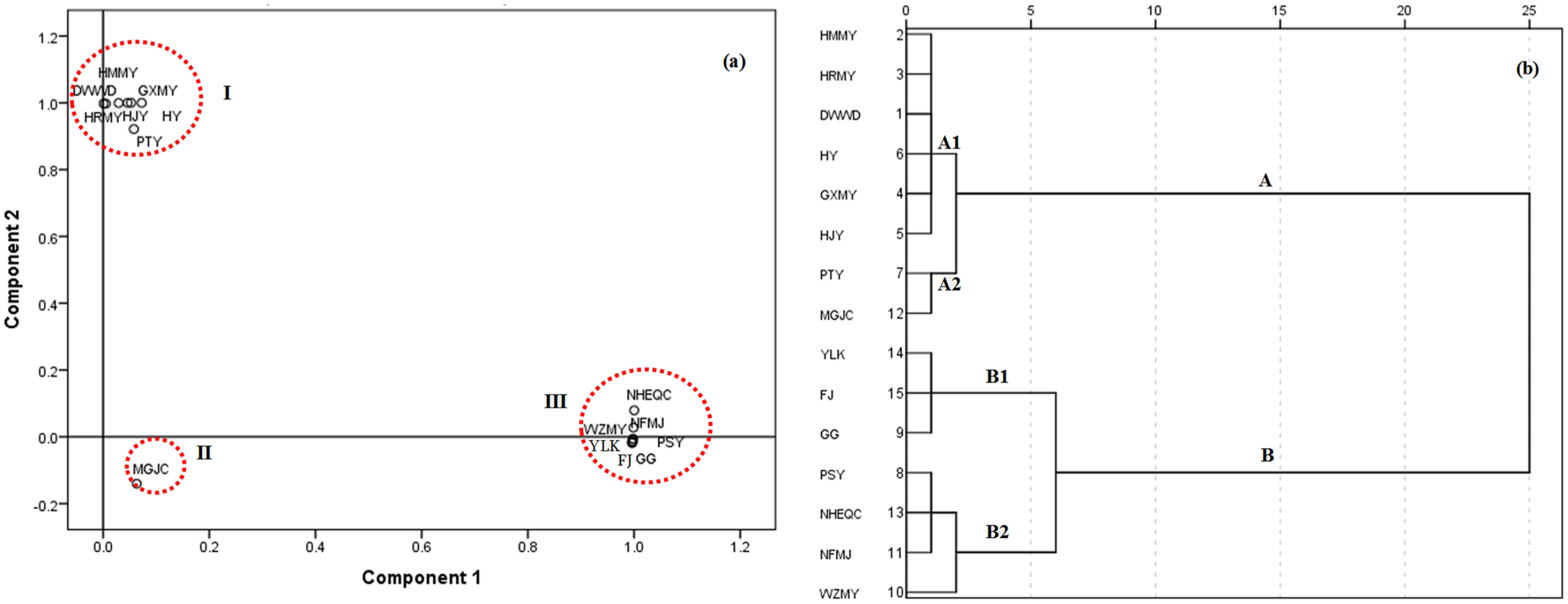
Classification of 15 citrus varieties. (A) 2D dimensional graphic of PCA scores of the 15 citrus cultivars by the 16 phenolic components, the 15 citrus cultivars were clustered into three groups (I, II and III); (B) HCA dendrogram of the 15 citrus cultivars by the 16 phenolic components, all of the tested samples were sorted into two main clusters (A and B) with two subgroups (A1, A2, B1 and B2) for each cluster.

For citrus, PCA was generated with two significant principal components (PCs) explaining 52.50% and 38.56% of the variance, respectively (Data S2). The CPV of the two PCs therefore explained 92.14% of the total variance. The resulting data was then plotted to produce a 2D dimensional graphic of PCA scores (Fig. 2A). In general, the 15 citrus cultivars were clustered into three groups: Samples 1–7 (Duweiwendan, Hongmianmiyou, Huangjinyou, Guangximiyou, Huangjinyou, Huyou and Putaoyou) in Table 1 as group I, sample 12 (Maogujucheng) as group II and the other seven samples as group III. All of the samples were sorted into two main clusters using HCA analysis (A and B, Fig. 2B). Samples 1–7 and 12 were in cluster A, whereas the other samples were in cluster B. Both cluster A and B were further divided into subgroup A1: Samples 1–6, subgroup A2: samples 7 and 11, subgroup B1: Samples 9, 14 and 15 and subgroup B2: samples 10, 11 and 13. Though there was some difference, significant overlap in the clustering of results between PCA and HCA was found. Generally, Cluster B included all the citrus cultivars in group III classified by PCA (Figs. 2A vs. 2B); samples 1–7 were always in one group (group I or cluster A). The difference in grouping sample 12 may be due to the PCA method only considering two main PCs, while HCA considering all of the components.

**Figure 3:**
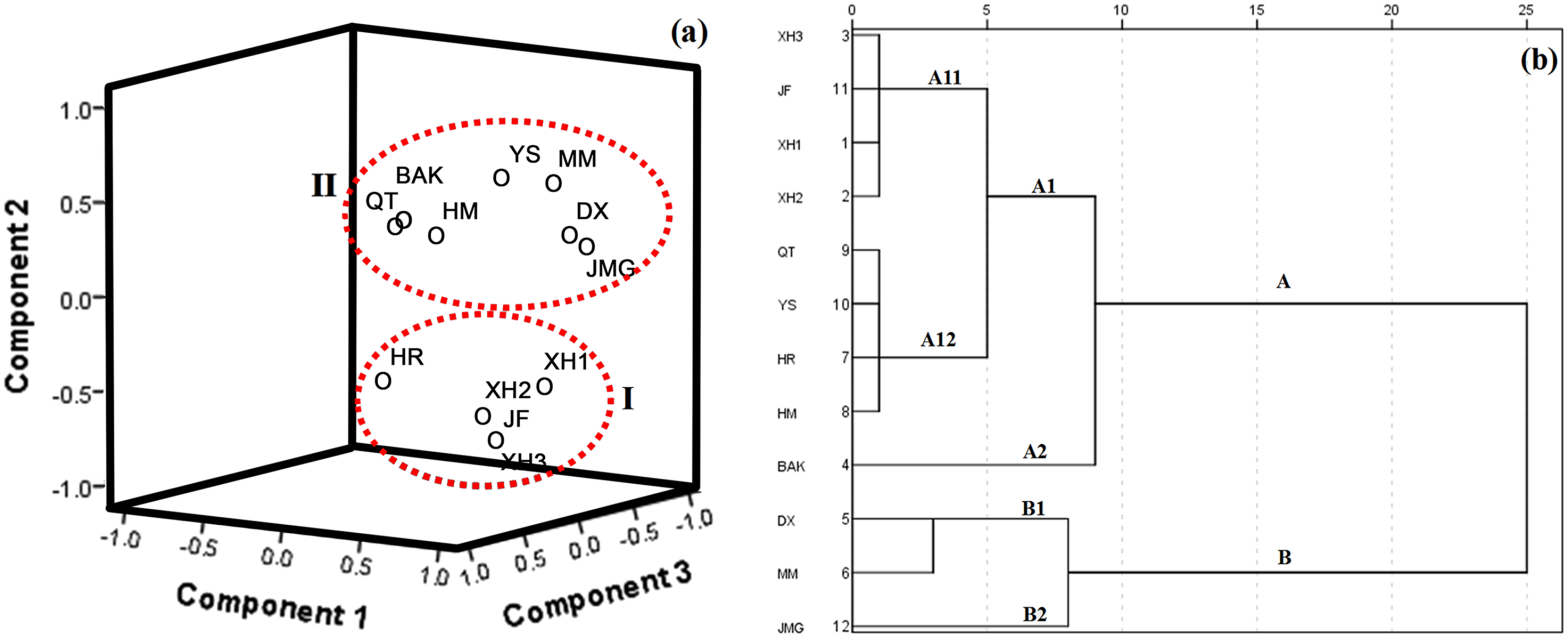
Classification of 12 grape varieties. (A) 3D dimensional graphic of PCA scores of the 12 grape cultivars by the 16 phenolic compounds, the 12 grape cultivars were also classified into two groups (I and II); (B) HCA dendrogram of the 12 grape cultivars by the 16 phenolic compounds, the 12 samples were grouped into two main clusters (A and B) with two subgroups (A1, A2, B1 and B2) for each cluster, and A1 has two subgroups (A11 and A12).

For grape, three significant PCs explaining 43.89%, 21.95% and 18.66% of the variance, respectively, corresponding to a CPV of 83.10% of the total variance were taken for further PCA analysis (Data S2). A 3D graphic of PCA scores was generated, which is shown in Fig. 3A. Generally, the 12 grape cultivars were also classified into two groups (Fig. 3A). XH1, XH2, XH3, JH and HR were in group I and the other grape cultivars were in group II. When HCA was used to cluster samples, the 12 samples were also grouped into two main clusters with two subgroups for each cluster (Fig. 3B). Though HCA clustered DX, MM and JMD in one group and the other cultivars in another group, both HCA and PCA classified XH1, XH2, XH3, JH and HR in one group, indicating a strong consistency between these two methods in evaluating similar patterns of plant cultivars belonging to the same genus.

## Discussion

In this study, HPLC method was used to verify that the established method has high sensitivity and good precision, which is sufficient to determine 17 compounds in citrus and grape. Peel extract rather than pulp extract was used due to its higher phenolic content in citrus (Sir Elkhatim, Elagib & Hassan, 2018). Flavonoids are classified into flavanone, flavonol, flavone, isoflavone, flavan-3-ols and anthocyanin, and hesperidin belongs to flavanone (Liew et al., 2018). The results showed that flavonoid hesperidin was the main flavonoid compound in all citrus samples, which was consistent with previous studies (Ernawita et al., 2017; Gomez-Mejia et al., 2019; Lou & Ho, 2017), where the authors concluded that hesperidin was a dominant flavonoid compound in citrus. Naringin (28.15–8,203.71 µg/g DW), quercetin (4.07–1,816.04 µg/g DW), nobiletin (not detectable (ND)-2,661.66 µg/g DW), tangeritin (ND-769.76 µg/g DW) and rutin (13.43–1,801.38 µg/g DW) were also abundant in citrus samples. These results were different from those reported by Zhang et al. (2014), in which naringin was an abundant compound. Among 16 samples, naringin was not detected in eight samples, while its concentrations were between 1,160 µg/mL and 13,610 µg/mL in the other eight samples (Zhang et al., 2014).

Generally, the concentrations of phenolic acids were lower than those of flavonoids, which is expected since methanol: DMSO (50:50, v/v) is more suitable for extracting flavonoids than phenolic acids, which has been reported by Magwaza et al. (2016). Our results differ from previous studies (Liew et al., 2018) which found that ferulic acid was the main phenolic acid in citrus peel extracts, and the abundance of phenolic acids in citrus varied in the following order: ferulic acid > caffeic acid > 4-hydroxybenzoic acid > protocatechuic acid. Zhang et al. (2014) also reported that ferulic acid was the most abundant phenolic acid in the peels of citrus fruits, with ferulic acid (1,613.34–7,780.17 µg/g DW) > caffeic acid (46.24–1,273.47 µg/g DW ) ∼ ρ-coumaric acid (154.55–834.77 µg/g DW) > sinapic acid (ND-342.84 µg/g DW). However, Xi et al. (2017) reported that caffeic acid was the main phenolic acid in the citrus species, with caffeic acid (9.31–741.4 μg/g DW) > chlorogenic acid (2.7–527.5 μg/g DW) > Gallic acid (ND–90.69 μg/g DW) > ferulic acid (ND-2.75 μg/g DW). In our study, the variation order of the phenolic acid contents observed was as follows: salicylic acid > benzoic acid > chlorogenic acid > ferulic acid > caffeic acid > ρ-coumaric acid > syringic acid > gallic acid. These differences resulted from the genetic difference of the citrus cultivars, and environmental and pedoclimatic factors (Zhang et al., 2014).

Similar to the flavonoid distribution in citrus, considerable differences in content of flavonoid compounds were detected among grape varieties. Findings in concentration difference among samples have been widely reported in the literatures (Colombo et al., 2019; Li & Sun, 2019; Tang et al., 2018), which was related to the variety of grape, environmental and pedoclimatic factors, and management of the vineyard. Despite that the order of abundance is different from the results by other authors, the abundance of rutin, quercetin, catechin and epicatechin in grape is the same (Tang et al., 2018). It is worth highlighting that the abundance of hesperidin in citrus has been widely reported (De Paula Menezes Barbosa, Ruviaro & Macedo, 2018; Gomez-Mejia et al., 2019), while it is rarely reported in grape. Our study provides an idea on the distribution of hesperidin in the peels of grapes. In addition, salicylic acid is also the most abundant phenolic acid in grapes. The difference between our results and the study by Liu et al. (2018), who mentioned that caffeic acid was the major phenolic acid in grape, was attributed to a more limited number of phenolic acid types determined by these authors.

Principal component analysis and HCA are powerful chemometric methods that have been widely used for classification studies (Shawky & Abou El Kheir, 2018). PCA involves unsupervised pattern recognition, which is the most frequently used method for reducing the dimensionality of numerical datasets in a multivariate space. It transforms the original set of variables to a new set of uncorrelated variables that are called PCs (Abegaz et al., 2019). PCA has been widely used for taxonomic discrimination, quality assessment or classification between plants from different geographic origins, and authenticity verification (for example, in Chinese herbal medicines) (Yan et al., 2020). Plotting the PCA scores highlighting the similarities and differences between groups. In order to map a PCA score, the initial eigenvalues were generated by inputting the average concentration of 16 determined phenolic components in the citrus or grape samples into SPSS software. PCs were selected to meet the requirement of cumulative percent variance (CPV) > 70–85% (Yi et al., 2015).

Hierarchical cluster analysis is a statistical method used for finding relatively homogeneous clusters of objects based on measured characteristics. Samples with the most similarities are clustered preferentially (Yan et al., 2020). To obtain the HCA dendrogram, the 16 phenolic compounds were set as variables, Ward’s method was selected as the cluster method, and squared Euclidean distance as the interval measurement (Yi et al., 2015; Zhao et al., 2017). PCA and HCA were used to analyze the chemical analysis of 15 citrus and 12 grape varieties. The results showed that citrus and grape were divided into two main groups by PCA and HCA with strong consistency.

## Conclusions

A highly precise and accurate HPLC-DAD method was herein developed and validated for the first time for simultaneous determination of 16 phenolic compounds in the peel of different citrus and grape cultivars, which verified our hypothesis. The method was eventually applied to quantify the phenolic constituents in the fruits of different citrus species and grape varieties to monitor their quality. Among the 16 compounds, hesperidin and salicylic acid were used as representative compounds for quality control of citrus and grape cultivars. In addition, chemometric analysis, including PCA and HCA, were performed to cluster 15 citrus and 12 grape cultivars. The quantitative data of the 16 phenolic compounds in citrus and grape samples were determined by the established HPLC protocol. The results revealed that citrus and grape were clustered into two main groups by PCA and HCA with strong consistency. This study provides information on the distribution of phenolic compounds in different citrus or grape cultivars in Fujian China, and gives a raw idea about the genetic relationship of these cultivars.

## Supplemental Information

10.7717/peerj.9083/supp-1 Supplemental Information 1 The contents of flavonoids and phenolic acid in the skin of the 15 different citrus cultivars and 12 grape cultivars analyzed in this study (µg/g DW) *.

10.7717/peerj.9083/supp-2 Supplemental Information 2 Principal Component Analysis of Citrus and Grape.

## Additional Information and Declarations

### Data Availability

The following information was supplied regarding data availability: The raw measurements are available in the Supplemental Files.
