# Supplementary material for: Simultaneous determination of phenolic metabolites in Chinese citrus and grape cultivars
Source: PeerJ. 2020 Jun 3;8:e9083. doi: 10.7717/peerj.9083 (PMC7275686; doi:10.7717/peerj.9083)
Supplement: Supplemental Information 2 [file peerj-08-9083-s002.docx]

**Citrus**

| **Total Variance Explained** | | | | | | |
| --- | --- | --- | --- | --- | --- | --- |
| Component | Initial Eigenvalues | | | Extraction of squared loadongs | | |
|  | Total | % of Variance | Cumulative % | Total | % of Variance | Cumulative % |
| 1 | 7.874 | 52.496 | 52.496 | 7.874 | 52.496 | 52.496 |
| 2 | 5.784 | 38.563 | 91.059 | 5.784 | 38.563 | 91.059 |
| 3 | .996 | 6.640 | 97.699 |  |  |  |
| 4 | .181 | 1.207 | 98.906 |  |  |  |
| 5 | .146 | .975 | 99.881 |  |  |  |
| 6 | .017 | .110 | 99.991 |  |  |  |
| 7 | .001 | .007 | 99.999 |  |  |  |
| 8 | .000 | .001 | 99.999 |  |  |  |
| 9 | 6.879E-5 | .000 | 100.000 |  |  |  |
| 10 | 1.046E-5 | 6.976E-5 | 100.000 |  |  |  |
| 11 | 6.748E-6 | 4.499E-5 | 100.000 |  |  |  |
| 12 | 4.497E-6 | 2.998E-5 | 100.000 |  |  |  |
| 13 | 8.180E-7 | 5.453E-6 | 100.000 |  |  |  |
| 14 | 3.368E-7 | 2.245E-6 | 100.000 |  |  |  |
| 15 | 9.451E-9 | 6.301E-8 | 100.000 |  |  |  |
| Extraction Method: Principal Component Analysis. | | | | | | |

**Grape**

| **Total Variance Explained** | | | | | | | |
| --- | --- | --- | --- | --- | --- | --- | --- |
| Component | Initial Eigenvalues | | | Extraction of squared loadongs | | | |
|  | Total | % of Variance | Cumulative % | Total | % of Variance | | Cumulative % |
| 1 | 5.705 | 43.888 | 43.888 | 5.705 | 43.888 | 43.888 | |
| 2 | 2.853 | 21.945 | 65.832 | 2.853 | 21.945 | 65.832 | |
| 3 | 2.425 | 18.657 | 84.489 | 2.425 | 18.657 | 84.489 | |
| 4 | .921 | 7.081 | 91.570 |  |  |  | |
| 5 | .853 | 6.560 | 98.130 |  |  |  | |
| 6 | .190 | 1.461 | 99.591 |  |  |  | |
| 7 | .024 | .182 | 99.773 |  |  |  | |
| 8 | .021 | .158 | 99.931 |  |  |  | |
| 9 | .005 | .040 | 99.971 |  |  |  | |
| 10 | .003 | .026 | 99.997 |  |  |  | |
| 11 | .000 | .003 | 100.000 |  |  |  | |
| 12 | 1.775E-16 | 1.365E-15 | 100.000 |  |  |  | |
| 13 | -1.066E-16 | -8.202E-16 | 100.000 |  |  |  | |
| Extraction Method: Principal Component Analysis. | | | | | | | |
